# Dimensional structure and internal consistency of the Brazilian Portuguese version of the Mistreatment of Women during Childbirth Questionnaire

**DOI:** 10.1590/1980-549720260036

**Published:** 2026-07-31

**Authors:** Emanuele Souza Marques, Andreas Bauer, Marília Arndt Mesenburg, Thaiza Dutra Gomes e Carvalho, Maria do Carmo Leal, Tatiana Henriques Leite

**Affiliations:** I Universidade do Estado do Rio de Janeiro, Institute of Social Medicine Hesio Cordeiro, Department of Epidemiology - Rio de Janeiro (RJ), Brazil.; II Universidade de São Paulo, School of Medicine, Department of Preventive Medicine - São Paulo (SP), Brazil.; III Fundação Oswaldo Cruz, National Institute of Women’s, Children’s, and Adolescents’ Health Fernandes Figueira - Rio de Janeiro (RJ), Brazil.; IV Fundação Oswaldo Cruz, National School of Public Health Sergio Arouca - Rio de Janeiro (RJ), Brazil.

**Keywords:** Obstetric violence, Questionnaires, Validation studies, Psychometrics, Factor analysis, Network analysis, Violência obstétrica, Questionários, Estudos de validação, Psicometria, Análise fatorial, Análise de rede

## Abstract

**Objective:**

This study aimed to evaluate the configural and metric structure, as well as internal consistency, of the Brazilian Portuguese adaptation of an instrument designed to assess mistreatment during childbirth.

**Methods:**

Data were drawn from the “Birth in Brazil” II*,* carried out in 2023/2024, comprising 1,881 women hospitalized for childbirth in the state of Rio de Janeiro, Brazil. Initially, confirmatory factor analysis was conducted to test the original dimensional structure. Due to an inadequate model, exploratory factor analysis was performed to reassess the factorial structure. Applying predefined criteria, items presenting cross-loadings or possible residual correlations were removed. The remaining items were subsequently evaluated using confirmatory factor analysis, and reliability was assessed using Cronbach’s α coefficients.

**Results:**

The final solution comprised 17 items distributed across four dimensions: verbal abuse; neglect and disrespect; stigma, discrimination and lack of privacy; and innappropriate vaginal examinations. The final model presented acceptable fit indices, satisfactory factorial definition, and no item redundancy. Most dimensions showed reasonable internal consistency, except the stigma, discrimination, and lack of privacydimension.

**Conclusion:**

The findings suggest that further psychometric evaluation is necessary to establish the instrument’s validity and reliability. Its use for assessing mistreatment during childbirth or obstetric violence in Brazil should be done with caution. Future psychometric studies using this instrument are recommended.

## RESUMO

**Objetivo:**

Este estudo teve como objetivo avaliar a estrutura configural e métrica, bem como a consistência interna, da adaptação para o português do Brasil de um instrumento destinado a avaliar maus-tratos durante o parto.

**Métodos:**

Utilizaram-se dados do estudo “Nascer no Brasil” II, realizado em 2023/24, que incluiu 1.881 mulheres hospitalizadas para parto no Rio de Janeiro, Brasil. Inicialmente, foi realizada uma Análise Fatorial Confirmatória (AFC) para testar a estrutura dimensional original. Devido à inadequação do modelo, realizou-se uma Análise Fatorial Exploratória (AFE) para reavaliar a estrutura fatorial. Aplicando critérios predefinidos, os itens que apresentaram cargas cruzadas ou possíveis correlações residuais foram removidos. Os itens restantes foram avaliados por meio de AFC; e a confiabilidade foi avaliada via coeficiente alfa de Cronbach.

**Resultados:**

A solução final compreendeu 17 itens distribuídos em quatro dimensões: abuso verbal; negligência e desrespeito; estigma, discriminação e falta de privacidade; e toques vaginais inadequados. O modelo final apresentou índices de ajuste aceitáveis, definição fatorial satisfatória e ausência de redundância de itens. A maioria das dimensões apresentou consistência interna razoável, com exceção da dimensão “estigma, discriminação e falta de privacidade”.

**Conclusão:**

Os resultados sugerem que é necessária uma avaliação psicométrica adicional para estabelecer a validade e a confiabilidade do instrumento. Seu uso para avaliar maus-tratos durante o parto ou violência obstétrica no Brasil deve ser feito com cautela. Recomenda-se estudos psicométricos futuros com este instrumento.

## INTRODUCTION

Mistreatment during childbirth in health facilities, despite being recognized as a public health concern and a violation of women’s rights, lacks a consensus-based term and definition^1^. This conceptual ambiguity poses challenges for measurement, as phenomena that are inconsistently defined cannot be reliably or validly assessed^2^ ^,^ ^3^.

The most widely used definition of mistreatment during childbirth was proposed by Bohren et al.^4^, who conceptualized it as comprising seven dimensions:

1. Physical abuse;
2. Sexual abuse;
3. Verbal abuse;
4. Stigma and discrimination;
5. Inappropriate health practices;
6. Poor communication with healthcare providers; and
7. Health system constraints and deficiencies.

This research group subsequently developed a set of items intended to operationalize these dimensions. The instrument was first implemented in a WHO-led multicountry study (Ghana, Guinea, Myanmar, and Nigeria), which found that over one-third of women experienced some form of mistreatment during childbirth in health facilities^5^. However, the empirical structure of the proposed item set did not fully align with the conceptual framework. Instead, the instrument was organized into five distinct dimensions: physical abuse (9 items), verbal abuse (13 items), neglect (4 items), stigma and discrimination (7 items), and vaginal examinations (4 items), totaling 37 items^6^. Note that the questionnaire also includes specific items addressing pain relief (2 items) and issues related to fluids, mobility, and the presence of a companion (6 items). However, these domains were not included in the psychometric evaluations conducted by the researchers who developed the instrument; they were analyzed separately only in the multicountry study (descriptive study)^5^.

Psychometric studies of this instrument remain limited. To date, only two studies - led by the researchers involved in its development - have assessed its measurement properties, yielding divergent results^7^ ^,^ ^8^. These discrepancies stem, in part, from differences in the item sets analyzed.

Berger et al.^7^ proposed a more concise version of the instrument covering three dimensions of mistreatment: interpersonal abuse (7 items), inappropriate conduct of examinations and procedures (3 items), and an unsupportive birth environment (12 items). Notably, several of these items were derived by merging two or more items from the original World Health Organization (WHO) study instrument. Similarly, Leslie et al.^8^ evaluated five dimensions: physical abuse (4 items), verbal abuse (10 items), failure to meet professional standards of care (10 items), poor rapport with healthcare workers (7 items), and health system conditions and constraints (7 items). Each of these dimensions was assessed using both full-length and brief formats, and again, some items reflected a combination of multiple original items.

Validity studies have focused on construct validity via hypothesis testing and on limited analyses of internal dimensional structure. Concerning configural and metric structure, Berger et al.^7^ employed preliminary exploratory analyses using principal components analysis, followed by exploratory and confirmatory factor analyses. Leslie et al.^8^, in contrast, utilized item response theory alongside confirmatory factor analysis. As each study used a different subset of items, comparisons of their findings to the original conceptual model must be interpreted with caution. Regarding reliability, Berger et al.^7^ and Leslie et al.^8^ evaluated internal consistency and obtained satisfactory results.

In light of the inconsistencies and methodological gaps identified in previous research, and as part of the ongoing cross-cultural adaptation process of the Mistreatment of Women during Childbirth Questionnaire, this study aimed to examine the configural and metric structure and to evaluate the internal consistency of the Brazilian Portuguese-adapted version of the instrument.

## METHODS

### Design, sampling, and participants

This study is based on subset data from hospital-based perinatal cohort *Nascer no Brasil II* (Birth in Brazil II), restricted to the state of Rio de Janeiro. The cohort comprised a baseline and two follow-up telephone interviews conducted at the second and fourth months postpartum. Data collection occurred between 2023 and 2024^9^.

The target population of the study comprised women admitted for childbirth (live birth or stillbirth) or abortion/miscarriage in hospitals that perform 100 or more deliveries per year, according to the Brazil Live Birth Information System (SINASC). Women with severe communication impairments (such as serious mental disorders, lack of fluency in Portuguese, or deafness) were excluded, as well as those with triplet or higher-order births and women discharged while still pregnant following an unconfirmed diagnosis of abortion^9^.

For this analysis, data collection was completed covering a total of 29 maternity hospitals observing the following eligibility criteria:

1. Women admitted for childbirth (live birth or stillbirth) in selected maternity hospitals in the state of Rio de Janeiro;
2. Who participated in the second follow-up of the study, conducted four months postpartum; and
3. Completed the questionnaire on mistreatment of women during childbirth in its entirety.

Based on these criteria, the final sample comprised 957 women, representing 50.9% of the original sample (n=1,881).

### Measurement tools

The Brazilian Portuguese-adapted version of the Mistreatment of Women during Childbirth Questionnaire encompasses five dimensions: physical abuse (7 items), verbal abuse (13 items), neglect (4 items), stigma and discrimination (6 items), and inappropriate vaginal examinations (4 items), totaling 34 items.

In the original version, the dimensions

1. Physical abuse and
2. Stigma and discrimination comprised nine and seven items, respectively.

The Brazilian Portuguese version presented seven and six items due to the merging of conceptually related items with very low frequency in previous empirical applications^5^. The items “During the observation period, was the woman slapped?” and “During the observation period, was the woman punched?” were merged into a single item in Portuguese (“*Algum profissional de saúde ou outro funcionário te deu um tapa ou soco?*”). Similarly, the items “During the observation period, was the woman physically tied to the bed (e.g., with linen or ropes)?” and “During the observation period, was the woman held down to the bed forcefully?” were combined into a single Portuguese item (“*Algum profissional de saúde ou outro funcionário te segurou à força, enforcou ou te amarrou na cama*?”).

In the stigma and discrimination dimension, the items “During the observation period, did the woman receive negative comments about her education or literacy level?” and “During the observation period, did the woman receive negative comments about her lower economic circumstances (e.g., poverty)?” were merged into a single item in Portuguese (“*Algum profissional de saúde ou outro funcionário fez comentários negativos sobre sua escolaridade ou situação financeira?*”). The original questionnaire proposed by Bohren et al.^6^ and the Brazilian Portuguese-adapted version are presented in the supplemental material (Table S1).

The cross-cultural adaptation process followed the framework proposed by Herdman et al.^10^. The assessment of conceptual and item equivalence for the Mistreatment of Women during Childbirth Questionnaire was conducted by a panel of three Brazilian researchers with doctoral-level training and studies in epidemiology, women’s health, and the violence area. Semantic equivalence was evaluated through two independent translations from English into Portuguese, performed by professional bilingual translators. The initial translation aimed to preserve the conceptual meaning of the items from the original version developed by Bohren et al.^6^. Subsequently, the same expert panel, composed of researchers fluent in English and with expertise in the construct addressed by the instrument, reviewed both translated versions and developed a synthesis version. This version was then pretested in a small number of participants to identify and resolve potential problems related to comprehension, wording, and cultural appropriateness of the items.

Additionally, a multi-thematic questionnaire was applied (at baseline and at the four-month postpartum). The multi-thematic questionnaire included variables related to demographic characteristics, socioeconomic status, and the current pregnancy.

### Data analysis

**Figure 1.**
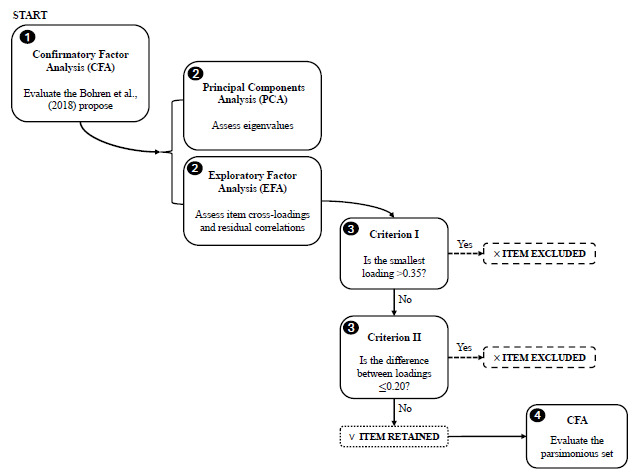
The psychometric analytical sequence.

Source: Elaborated by the authors.

The analytical sequence is detailed in Figure 1. Data analysis was conducted in four steps. Initially, the 27-item, four-factor model proposed by Bohren et al.^6^ was evaluated using confirmatory factor analysis (CFA)^11^. To identify the presence of possible anomalies, modification indices (MI; based on univariate Lagrange multipliers) and expected parameter changes (EPC)^11^ ^,^ ^12^ were examined. The MI indicates the potential decrease in model chi-square if a given parameter is freely estimated, while the EPC provides an estimate of the expected change in the parameter value^11^. Factorial correlations were assessed, with values >0.80 indicating moderate concern and >0.85 suggesting a potential violation of factor discriminant validity^11^.

In light of the anomalies identified during step 1, an exploratory approach was adopted in the second step. A principal components analysis (PCA) was conducted to determine the number of components with eigenvalues greater than 1.0^13^. Based on these findings, an exploratory factor analysis (EFA) was fitted to assess item cross-loadings and residual correlations. The analysis used Geomin oblique rotation^11^.

In a third step, a parsimonious set of items was selected to ensure the absence of factorial ambiguity.

For each item in the first EFA solution (step 2), the two highest factor loadings were identified and sequentially evaluated according to predefined criteria^14^:

1. Items with the smallest loading >0.35 were excluded; and
2. Items with a difference ≤0.20 were also excluded. After each iteration, a new EFA was conducted with the remaining items, and the criteria were reapplied.

This iterative procedure continued until no items met the exclusion criteria.

The final step involved returning to a CFA using the findings obtained from the previous steps. Dimensionality was reassessed using MI and respective EPC. All analyses employed probit link functions and polychoric correlation matrices, using the robust diagonally weighted least squares estimator (weighted least squares mean and variance; WLSMV), which is appropriate for categorical data and can accommodate missing values^15^.

Model fit was evaluated using the root mean square error of approximation (RMSEA), comparative fit index (CFI), and Tucker-Lewis index (TLI). RMSEA accounts for model complexity and degrees of freedom, with values <0.06 indicating good fit and values >0.10 suggesting poor fit^11^. The 90% upper confidence limit (UCL) for the RMSEA was also considered^11^ ^,^ ^16^. CFI and TLI values >0.95 were considered indicative of an acceptable model fit. All factor analyses were performed in Mplus version 8.11^17^.

To assess internal consistency, Cronbach’s α coefficients and their one-sided 95% lower confidence limits were calculated. According to Nunnally and Bernstein^18^, α≥0.70 were deemed acceptable for early-stage scale development. Internal consistency analyses and descriptive analyses of the study population were performed using Stata version 19.0^19^ (Figure 1).

### Ethics aspects

This study was approved by the Brazilian National Research Ethics Commission (*Comissão Nacional de Ética em Pesquisa* - CONEP), with Certificate of Presentation for Ethical Consideration (CAAE): 21633519.5.0000.5240, as well as by the local Research Ethics Committees of the participating hospitals, whenever required. Before each interview, informed consent was obtained from the postpartum women following a thorough reading of the Free and Informed Consent Form.

## Data availability statement:

The entire dataset supporting the results of this study is available upon request from the corresponding author.

## RESULTS

The study population comprised women aged between 14 and 49 years, with a mean age of 27.8 years and a standard deviation of 6.5 years. The majority self-identified as white (29.3%), had completed high school (40.2%), and reported having a partner (88.7%). Most women did not go into labor (52.4%), underwent cesarean delivery (57.0%), and gave birth in public healthcare facilities (76.3%). The prevalence rates of mistreatment during childbirth were physical abuse (0.3%), verbal abuse (21.5%), and neglect (28.5%). Additionally, 6.9% of participants reported experiencing stigma and discrimination, while 46.0% reported being subjected to inappropriate vaginal examinations.

Table 1 presents the results of the CFA concerning the four-factor model proposed by Bohren et al.^6^. Seven items from the physical abuse dimension were excluded from this analysis due to the low frequency of most items’ responses. The RMSEA was acceptable, but the CFI and TLI indices presented borderline values (0.95>x>0.90). Only 2 items (ed1 and tv3) showed residuals (δ_i_) >0.70. Some MI with standardized EPC≥0,20 indicating cross-loadings were identified.

**Table 1.** Analysis of the dimensional structure of the Brazilian Portuguese-adapted version of the Mistreatment of Women during Childbirth Questionnaire using confirmatory factor analysis.

Items		Model 4-factor CFA				
		λ_i(1)*_	λ_i(2)_	λ_i(3)_	λ_i(4)_	δ_i†_
p1	Shouted or screamed at you	0.80				0.36
p2	Insulted	0.84				0.29
p3	Scolded	0.85				0.27
p4	Mocked	0.78				0.40
p5	Receive negative comments about her physical appearance	0.68				0.54
p6	Receive negative comments about the baby’s physical appearance	0.66				0.56
p7	Receive comments about her sexual activity	0.77				0.40
p8	Threatened with use of a medical procedure	0.63				0.61
p9	Threatened with physical violence	0.82				0.33
p10	Threatened that if she does not comply, her or her baby will have a poor outcome	0.90				0.20
p11	Threatened with withholding care from her or her baby	0.75				0.44
p12	Hissed	0.92				0.16
p13	Blamed for her or her baby’s poor health or outcomes	0.74				0.45
ed1	Receive negative comments about her ethnicity or race		0.53			0.71
ed2	Receive negative comments about her religion		0.74			0.45
ed3	Receive negative comments about her age		0.79			0.38
ed4	Receive negative comments about her marital status		0.66			0.57
ed5	Receive negative comments about her education or lower economic circumstances		0.84			0.29
ed6	Receive negative comments about her health condition		0.77			0.40
n1	Felt ignored			0.97		0.06
n2	Felt neglected			0.95		0.10
n3	Felt that my presence was a nuisance			0.97		0.06
n4	Had to wait for long periods of time before I was attended			0.77		0.41
tv1	Information about vaginal examination is needed				0.79	0.38
tv2	Obtain permission of the woman before the vaginal examination				0.93	0.14
tv3	Discuss the woman’s private health information in a way that others could hear				0.28	0.92
tv4	Vaginal examination in a way that others could see her genitalia				0.72	0.48
RMSEA		0.016 (0.011; 0.021)^‡^				
CFI		0.936				
TLI		0.930				

CFA: confirmatory factor analysis; RMSEA: Root Mean Square Error of Approximation; CFI: Comparative Fit Index; TLI: Tucker-Lewis Index.

*Loadings (standardized); ^†^Measurement errors (uniqueness); ^‡^90% confidence intervals.

Source: Prepared by the authors based on the study data.

Considering the results of the first step, exploratory analyses were performed. The preliminary PCA indicated 12 eigenvalues >1.0. An analysis of the two-, three-, and four-factor solutions obtained through EFA revealed that all models included at least one item with a factor loading greater than one, which may be attributed to the presence of highly correlated items. The presence of cross-loadings across all tested solutions (Table 2) should be highlighted. In the four-factor solution, cross-loadings were identified for items p4, p5, ed4, n3, and tv4. In the subsequent EFA, items p8 [λi_(Factor 1)_: 0.417 and λi_(Factor 2)_: 0.361], p9 [λi_(Factor 1)_: 0.594 and λi_(Factor 2)_: 0.435], ed5 [λi_(Factor 1)_: 0.627 and λi_(Factor 2)_: 0.450], and ed6 [λi_(Factor 1)_: 0.451 and λi_(Factor 2)_: 0.499] were excluded. In the third EFA, only item p12 [λi_(Factor 1)_: 0.588 and λi_(Factor 2)_: 0.460] met the exclusion criteria. After removing item p12, a new EFA was conducted, and no additional items met the algorithm criteria for exclusion.

**Table 2.** Analysis of the dimensional structure of the Brazilian Portuguese-adapted version of the Mistreatment of Women during Childbirth Questionnaire using exploratory factor analysis.

	Model 2-factors			Model 3-factors				Model 4-factors				
	λ_i(1)*_	λ_i(2)_	δ_i†_	λ_i(1)*_	λ_i(2)_	λ_i(3)_	^†^	λ_i(1)*_	λ_i(2)_	λ_i(3)_	λ_i(4)_	δ_i†_
p1	0.81	-0.01	0.34	0.76	0.16	-0.09	0.32	0.83	0.17	-0.08	-0.16	0.22
p2	0.84	0.00	0.29	0.83	0.10	-0.04	0.25	0.57	0.29	0.31	-0.08	0.22
p3	0.78	0.13	0.35	0.83	-0.00	0.09	0.28	0.67	0.11	0.28	0.05	0.25
p4	0.76	-0.13	0.43	0.83	0.01	-0.19	0.33	0.40	0.26	0.41	-0.18	0.33
p5	0.70	-0.16	0.51	0.65	0.18	-0.07	0.48	0.03	0.49	0.57	-0.04	0.34
p6	0.70	-0.45	0.40	0.36	0.61	-0.00	0.32	0.22	0.72	0.10	0.03	0.30
p7	0.73	0.14	0.41	0.73	0.03	0.11	0.40	0.70	0.02	0.17	0.05	0.36
p8	0.61	0.05	0.62	0.69	-0.32	0.01	0.59	0.06	0.07	0.66	0.04	0.50
p9	0.84	-0.25	0.30	0.42	0.66	0.20	0.10	0.48	0.73	-0.03	0.17	0.07
p10	0.86	0.12	0.21	0.89	-0.04	0.05	0.21	0.92	-0.17	0.08	0.01	0.14
p11	0.76	-0.02	0.43	0.68	0.22	-0.05	0.38	1.07	0.02	-0.45	-0.02	0.08
p12	0.84	0.16	0.23	0.91	-0.07	0.09	0.17	0.86	-0.13	0.20	0.05	0.11
p13	0.78	-0.16	0.41	0.97	0.29	-0.15	0.33	0.61	0.33	0.07	-0.21	0.30
ed1	0.54	-0.65	0.39	-0.01	0.88	-0.14	0.21	-0.02	0.90	-0.12	-0.15	0.17
ed2	0.70	-0.49	0.38	0.21	0.74	0.05	0.27	0.29	0.76	-0.12	0.03	0.27
ed3	0.68	0.10	0.51	0.63	0.20	0.10	0.43	0.72	0.14	0.02	0.52	0.40
ed4	0.50	0.39	0.53	0.44	0.00	0.48	0.49	-0.15	0.47	0.63	0.09	0.14
ed5	0.79	-0.41	0.31	0.36	0.59	0.11	0.33	0.34	0.65	0.03	-0.06	0.33
ed6	0.74	-0.15	0.46	0.61	0.32	-0.03	0.39	0.18	0.59	0.39	-0.06	0.27
n1	0.91	0.04	0.15	1.02	-0.56	-0.06	0.11	0.36	-0.22	0.76	0.06	0.09
n2	0.91	0.08	0.14	1.04	-0.72	0.01	-0.02	0.28	-0.29	0.86	0.03	0.00
n3	0.79	0.20	0.28	0.93	-0.32	0.08	0.23	0.50	-0.06	0.56	0.06	0.20
n4	0.67	0.07	0.54	0.80	-0.37	0.01	0.44	0.06	0.09	0.77	0.03	0.32
tv1	0.03	0.73	0.46	-0.02	0.04	0.78	0.39	0.04	0.16	0.09	0.82	0.30
tv2	0.01	1.06	-0.12	-0.00	-0.03	10.03	-0.05	0.27	-0.02	-0.01	0.96	0.03
tv3	0.55	-0.12	0.71	0.33	0.44	-0.02	0.59	-0.01	0.63	0.25	-0.02	0.49
tv4	0.11	0.60	0.61	0.06	0.11	0.65	0.55	0.43	-0.04	-0.17	0.62	0.49
RMSEA	0.017 (0.011; 0.022) ^‡^				0.012 (0.000; 0.018) ^‡^				0.010 (0.000; 0.017) ^‡^			
CFI	0.937				0.973				0.982			
TLI	0.926				0.965				0.975			

RMSEA: Root Mean Square Error of Approximation; CFI: Comparative Fit Index; TLI: Tucker-Lewis Index.

*Loadings (standardized); ^†^Measurement errors (uniqueness); ^‡^90% confidence intervals.

Source: Prepared by the authors based on the study data.

Table 3 presents the results of the final analytical step, in which the CFA was applied to the most parsimonious model derived from the previous stage. All factor loadings exceeded 0.40, ranging from 0.673 to 0.959, with 13 loadings ≥0.80. Five MI and standardized EPC values ≥0.20 were identified.

**Table 3.** Confirmatory factor analysis applying the most parsimonious model obtained in exploratory factor analysis of the Brazilian Portuguese-adapted version of the Mistreatment of Women during Childbirth Questionnaire.

Dimension	Items	Model 4-factor CFA				
		λ_i(1)*_	λ_i(2)_	λ_i(3)_	λ_i(4)_	δ_i†_
Verbal abuse	p1	0.85				0.27
	p2	0.95				0.09
	p3	0.80				0.36
	p10	0.85				0.27
	p11	0.91				0.18
	p13	0.76				0.42
Neglect and disrespect	p7		0.81			0.34
	ed3		0.66			0.57
	n1		0.96			0.08
	n2		0.96			0.09
	n4		0.77			0.42
Stigma, discrimination, and lack of privacy	p6			0.85		0.28
	ed1			0.67		0.55
	ed2			0.85		0.28
	tv3			0.94		0.11
Inappropriate vaginal examinations	tv1				0.84	0.30
	tv2				0.94	0.11
RMSEA		0.039 (0.033; 0.044)^‡^				
CFI		0.932				
TLI		0.918				

CFA: confirmatory factor analysis; RMSEA: Root Mean Square Error of Approximation; CFI: Comparative Fit Index; TLI: Tucker-Lewis Index.

*Loadings (standardized); ^†^Measurement errors (uniqueness); ^‡^90% confidence intervals.

Source: Prepared by the authors based on the study data.

The final solution comprised 17 items distributed across four dimensions. Dimension 1 included items p1, p2, p3, p10, p11, and p13 and retained the original label “verbal abuse”. Dimension 2 comprised items p7, ed3, n1, n2, and n3 and was renamed “neglect and disrespect”. Dimension 3 included items p6, ed1, ed2, and tv3, and was renamed “stigma, discrimination, and lack of privacy”. Finally, Dimension 4, composed of only items tv1 and tv2, retained the original label “inappropriate vaginal examinations”.

Cronbach’s α coefficient for the original dimensions proposed by Bohren et al.^6^ - verbal abuse; neglect; stigma and discrimination; and vaginal examinations scales - were 0.77, 0.81, 0.30, and 0.62, respectively. Cronbach’s α coefficient for the four dimensions of the final model tested - verbal abuse; neglect and disrespect; stigma, discrimination, and lack of privacy; and vaginal examinations scales - were 0.70, 0.76, 0.22, and 0.67, respectively. These results indicate a satisfactory internal consistency for most dimensions, except the stigma, discrimination, and lack of privacy dimension, which presented low reliability.

## DISCUSSION

The Mistreatment of Women during Childbirth Questionnaire is an important tool for assessing mistreatment/obstetric violence during childbirth and has been widely used in epidemiological studies conducted in Africa by WHO. However, the psychometric evaluation of this instrument is recent and still limited. This study aimed to revisit the original dimensional structure proposed by Bohren et al.^6^ and contribute to this gap by analyzing the psychometric properties of the Brazilian Portuguese adaptation of the instrument, thereby broadening the debate regarding the validity of the original version and its cross-cultural adaptations.

Based on the two validation studies conducted by the original authors, the instrument showed reasonable levels of reliability^7^ ^,^ ^8^. In this study, some of the originally proposed dimensions showed low levels of internal consistency (especially the stigma and discrimination dimension; 𝛼=0.30). On the other hand, most dimensions of the final model presented acceptable internal consistency.

The dimensional structures identified in those studies diverged^7^ ^,^ ^8^, likely due to differences in the dimensions and items analyzed. Similarly, the final model identified in the present study differs from those previously proposed.

The final model comprised four dimensions:

1. Verbal abuse;
2. Neglect and disrespect;
3. Stigma, discrimination, and lack of privacy; and
4. Inappropriate vaginal examinations.

Overall, this structure presented some dimensions similar to the original ones but encompassed distinct items. Note that the final model has conceptual coherence and acceptable psychometric performance (high factor loadings and no redundancy). The verbal abuse dimension grouped items predominantly related to humiliating, threatening, or disrespectful verbal interactions. The inappropriate vaginal examinations dimension encompassed items specifically related to informed communication, consent, and privacy during vaginal examinations. The neglect and disrespect dimension deserves attention. This factor combined items related both to omission of care (e.g., feeling ignored or neglected) and disrespectful interactions. Likewise, the stigma, discrimination, and lack of privacy dimension presented items reflecting discriminatory treatment and violations of privacy. It is noteworthy that, in the additional iterative EFA, ten cross-loadings (p4, p5, p8, p9, p12, ed4, ed5, ed6, n3, and tv4) were identified. These findings suggest conceptual overlap between some domains of mistreatment of women during childbirth, which is expected given the multidimensional and interrelated nature of abusive and disrespectful care practices. Despite these refinements, some dimensions, particularly the stigma, discrimination, and lack of privacy dimension, continued to have low internal consistency.

This finding may reflect the heterogeneity and low frequency of some discriminatory and privacy-related experiences, as well as the limited number of items retained in this dimension. Further psychometric studies covering the set of items in the original proposal are needed in order to better assess the behavior of these items and the instrument as a whole.

An additional point that should be highlighted is the exclusion or aggregation of rare items, particularly the f2, f6, and ed5 items, as well as the exclusion of the physical violence dimension in this study. From a psychometric perspective, items with very low prevalence may contribute to unstable estimates, sparse response distributions, and difficulties in identifying reliable factorial structures. However, the exclusion of such items may also have important conceptual and ethical implications, as severe forms of mistreatment, although few are prevalent, represent relevant violations of women’s rights during childbirth care. Thus, decisions regarding the removal or combination of low-frequency items should not rely exclusively on statistical criteria but should also consider their theoretical and substantive relevance. In the present study, the exclusion of some items was guided both by their low occurrence in previous multicountry studies ^5^ and by the impossibility of carrying out the proposed psychometric analyses.

The findings of this study should be interpreted in light of its limitations and strengths. First, the Brazilian Portuguese-adapted version of the instrument combined certain items from the original version (e.g., f2, f6, ed5). Future research is needed to investigate the implications of using both the combined and disaggregated versions of these items in order to better understand their contribution to the instrument’s performance. It is important to highlight that, given the instrument’s length, this semantic merging may help reduce administration time, an important issue in large-scale epidemiological studies. Another limitation concerns the partial use of the original instrument. Due to the low endorsement of items related to the physical violence dimension, it was not possible to assess the psychometric properties of the full instrument. Similarly, due to estimation problems, some items had to be excluded from the network analysis. The need for large-scale national studies that allow for a comprehensive evaluation of the entire instrument, including psychometric properties not assessed in this study, such as discriminant factorial validity and scalar structure, should be emphasized. Among the strengths of this research, it is noteworthy that this is the first study to evaluate the psychometric properties of a cross-culturally adapted version of the instrument using data from an epidemiological study.

The results indicate that further psychometric evaluation is needed to establish the instrument’s validity and reliability. Accordingly, its use for assessing mistreatment during childbirth or obstetric violence in Brazil should be addressed with caution. Its application should be encouraged, as it expands the potential for psychometric studies and enhances comparability with international findings, particularly when contrasted with other instruments currently available to measure this phenomenon.

Although the present study proposes a reduced version of the instrument, characterized by lower item redundancy and improved factorial definition, it is suggested that future studies also consider using the originally proposed item set, given the still limited number of psychometric evaluations available. This more conservative approach may help preserve comparability with previous studies and support cumulative evidence building regarding the instrument’s performance.

Future analyses using nationally representative data, encompassing broader social and regional contexts and larger sample sizes, are essential to confirm, refine, and expand these findings. Continued refinement of instruments such as this one may contribute to more accurate measurement of mistreatment during childbirth in epidemiological research.

## Supplementary Material


