# Supplementary material for: Dimensional structure and internal consistency of the Brazilian Portuguese version of the Mistreatment of Women during Childbirth Questionnaire
Source: Rev Bras Epidemiol. 2026 Jul 31;29:e260036. doi: 10.1590/1980-549720260036 (PMC13427216; doi:10.1590/1980-549720260036)
Supplement: Supplementary file 1 [file 1980-5497-rbepid-29-e260036-sppl.docx]

Table S1: English (original) and Brazilian Portuguese adapted version of the mistreatment of women during childbirth questionnaire

| **Dimension** | **Item** | **Bohren et al. (2018)** | **Brazilian Portuguese adapted version** |
| --- | --- | --- | --- |
| **Physical abuse** | f1 | During the observation period, was the woman pinched? | Algum profissional de saúde ou outro funcionário te beliscou? |
|  | f2 | During the observation period, was the woman slapped? | Algum profissional de saúde ou outro funcionário te deu um tapa ou soco? |
|  |  | During the observation period, was the woman punched? |  |
|  | f3 | During the observation period, was the woman kicked? | Algum profissional de saúde ou outro funcionário te chutou? |
|  | f4 | During the observation period, was the woman hit with an instrument? | Algum profissional de saúde ou outro funcionário te bateu com algum objeto? |
|  | f5 | During the observation period, was the woman gagged (something put across or in her mouth to prevent her from speaking or making noise)? | Algum profissional de saúde ou outro funcionário te amordaçou ou colocou a mão na sua boca para impedir você de falar ou fazer qualquer barulho? |
|  | f6 | During the observation period, was the woman physically tied to the bed (eg: with linen or ropes)? | Algum profissional de saúde ou outro funcionário te segurou à força, enforcou ou te amarrou na cama? |
|  |  | During the observation period, was the woman held down to the  bed forcefully? |  |
|  | f7 | During the observation period, was the woman given forceful downward pressure directly onto women's abdomen (i.e. fundal pressure)? | Na hora do parto, alguém apertou/subiu na sua barriga para ajudar a saída do bebê? |
| **Psychological abuse** | p1 | During the observation period, was the woman shouted or screamed at? | Algum profissional de saúde ou outro funcionário gritou ou berrou com você? |
|  | p2 | During the observation period, was the woman insulted? | Algum profissional de saúde ou outro funcionário ofendeu ou xingou você? |
|  | p3 | During the observation period, was the woman scolded? | Algum profissional de saúde ou outro funcionário te repreendeu ou deu uma bronca? |
|  | p4 | During the observation period, was the woman mocked? | Algum profissional de saúde ou outro funcionário debochou de você? |
|  | p5 | During the observation period, did the woman receive negative comments about her physical appearance (including her weight, genitalia, cleanliness or other aspects of a woman's body)? | Algum profissional de saúde ou outro funcionário fez comentários negativos sobre sua aparência física (como seu peso, partes íntimas, higiene ou outras partes do seu corpo)? |
|  | p6 | During the observation period, did the woman receive negative comments about the baby's physical appearance (including his/her appearance, sex, or other aspects of the baby)? | Algum profissional de saúde ou outro funcionário fez comentários negativos sobre a aparência física do seu bebê (como a fisionomia, o sexo ou outros aspectos do bebê)? |
|  | p7 | During the observation period, did the woman receive comments about her sexual activity? | Algum profissional de saúde ou outro funcionário fez comentários negativos sobre sua vida sexual? Por exemplo: na hora de fazer não chorou, não chamou a mãe. |
|  | p8 | During the observation period, was the woman threatened with use of a medical procedure (such as episiotomy, caesarean section or other procedure)? | Algum profissional de saúde ou outro funcionário ameaçou a fazer algum procedimento médico que você não queria (como, por exemplo, corte na vagina, parto normal ou cesariana, uso de fórceps ou outros)? |
|  | p9 | During the observation period, was the woman threatened with physical violence? | Algum profissional de saúde ou outro funcionário ameaçou te machucar, te bater, chutar, dar tapa, soco, enforcar, amordaçar, amarrar ou outra forma de abuso físico? |
|  | p10 | During the observation period, was the woman threatened that if she does not comply, her or her baby will have a poor outcome? | Algum profissional de saúde ou outro funcionário da maternidade/hospital ameaçou você dizendo que se não obedecesse, você e seu bebê teriam problemas? |
|  | p11 | During the observation period, was the woman threatened with withholding care from her or her baby? | Algum profissional de saúde ou outro funcionário da maternidade/hospital ameaçou não cuidar ou parar de cuidar de você ou de seu bebê? |
|  | p12 | During the observation period, was the woman hissed at? | Algum profissional de saúde ou outro funcionário da maternidade/hospital bufou/resmungou com você durante o tempo que ficou no hospital? |
|  | p13 | During the observation period, was the woman blamed for her or her baby's poor health or outcomes? | Algum profissional de saúde ou outro funcionário te culpou por alguma coisa que tenha acontecido como você ou seu bebê durante a sua estadia no hospital? |
| **Stigma and discrimination** | ed1 | During the observation period, did the woman receive negative comments about her ethnicity or race? | Algum profissional de saúde ou outro funcionário da maternidade/hospital fez comentário negativos sobre a sua raça, cor de pele ou cultura? |
|  | ed2 | During the observation period, did the woman receive negative comments about her religion? | Algum profissional de saúde ou outro funcionário fez comentários negativos sobre a sua religião? |
|  | ed3 | During the observation period, did the woman receive negative comments about her age? | Fez algum comentário negativo sobre sua idade? Por exemplo, ser muito nova ou muito velha. |
|  | ed4 | During the observation period, did the woman receive negative comments about her marital status? | Algum profissional de saúde ou outro funcionário fez algum comentário negativo sobre você ter ou não um companheiro? |
|  | ed5 | During the observation period, did the woman receive negative comments about her education or literacy level? | Algum profissional de saúde ou outro funcionário fez comentários negativos sobre sua escolaridade ou situação financeira? |
|  |  | During the observation period, did the woman receive negative comments about her lower economic circumstances (e.g. poverty)? |  |
|  | ed6 | During the observation period, did the woman receive negative comments you regarding your HIV status? | Algum profissional de saúde ou outro funcionário fez comentários negativos considerando a sua situação de saúde (como ter diabetes, hipertensão, obesidade, covid, sífilis, ou outros problemas de saúde)? |
| **Neglect** | n1 | During my time in hospital for childbirth I felt ignored by the health workers or staff? | Você se sentiu ignorada pelos profissionais de saúde ou outros funcionários? |
|  | n2 | During my time in hospital for childbirth I felt neglected by the health workers or staff? | Você se sentiu abandonada pelos profissionais de saúde ou outros funcionários? |
|  | n3 | During my time in hospital for childbirth I felt that my presence was a nuisance for the health workers or staff? | Você sentiu que sua presença era um incômodo para os profissionais de saúde ou outros funcionários? |
|  | n4 | During my time in hospital for childbirth I had to wait for long periods of time before I was attended by health workers? | Durante sua estadia na maternidade/hospital você precisou esperar por longos períodos de tempo antes de ser atendida por um profissional de saúde? |
| **Vaginal examinations** | tv1 | Before giving a vaginal examination, did the staff inform the woman of why a vaginal examination is needed? | O profissional de saúde explicou a você por que o exame vaginal era necessário? |
|  | tv2 | Did the staff member obtain permission of the woman before the vaginal examination? | O profissional de saúde pediu sua permissão antes de fazer o exame vaginal? |
|  | tv3 | Did a staff member discuss the woman's private health information in a way that others (non-medical staff, other patients or other patients' family members) could hear? | Algum profissional de saúde ou outro funcionário falou informações confidenciais sobre a sua saúde de forma que outras pessoas pudessem ouvir? |
|  | tv4 | Did a staff member conduct a vaginal examination in a way that others (patients, visitors, non-medical staff) could see her genitalia? | Os exames vaginais foram feitos de forma privada/com privacidade (de um jeito que outras pessoas não pudessem ver)? |
